# Examining the predictive utility of suicidal ideation characteristics in relation to real‐time monitoring of suicidal ideation and suicide attempts at follow‐up

**DOI:** 10.1111/sltb.13103

**Published:** 2024-06-18

**Authors:** Megan L. Rogers, Melanie L. Bozzay, Christopher D. Hughes, Heather T. Schatten, Michael F. Armey

**Affiliations:** ^1^ Department of Psychology Texas State University San Marcos Texas USA; ^2^ The Ohio State University Wexner Medical Center Columbus Ohio USA; ^3^ Butler Hospital Providence Rhode Island USA; ^4^ Alpert Medical School of Brown University Providence Rhode Island USA

**Keywords:** ecological momentary assessment, perceived control, suicidal ideation, thought characteristics

## Abstract

**Introduction:**

Several characteristics of suicidal ideation, including frequency, duration, perceived controllability, and intensity, have been identified. The present study examined whether these characteristics of baseline suicidal ideation uniquely predicted (1) the severity, variability, and frequency of suicidal ideation assessed through real‐time monitoring; and (2) suicide attempts at 3‐week and 6‐month follow‐up among recently discharged psychiatric inpatients.

**Methods:**

A sample of 249 adults (*M*_age_ = 40.43, 55.1% female, 91.4% White) completed a baseline assessment of their suicidal ideation characteristics during psychiatric hospitalization, five daily ecological momentary assessments (EMA) for 21 days following discharge, and follow‐up assessments of suicide‐related outcomes at 3‐week and 6‐month follow‐up.

**Results:**

Perceived controllability of suicidal thoughts was uniquely associated with the variability of EMA‐assessed suicidal ideation and the presence of suicide attempts at 3‐week, but not 6‐month follow‐up. No other characteristic of baseline suicidal ideation was uniquely associated with EMA‐assessed suicidal ideation or the presence of suicide attempts at follow‐up.

**Conclusions:**

Given links between the perceived controllability of suicidal ideation and (1) momentary variability of suicidal ideation and (2) suicide attempts over the subsequent 3 weeks, perceived controllability of suicidal thinking may be a useful marker of short‐term risk that may be malleable to clinical intervention.

## INTRODUCTION

Suicide is a global public health concern, resulting in nearly 800,000 deaths (World Health Organization [WHO], 2021), approximately 25 suicide attempts for every death by suicide (Centers for Disease Control and Prevention [CDC], 2022), and countless individuals experiencing suicidal thoughts each year (CDC, 2022). Improvements in our understanding and assessment of the processes underlying suicide risk may aid in preventing and treating suicide; however, traditionally examined risk factors, including self‐injurious thoughts and behaviors (Ribeiro et al., 2016), only modestly predict future suicidal thoughts and behaviors (Franklin et al., 2017). When transitions from suicidal ideation to suicide attempts do occur, they tend to occur within a year of onset of suicidal ideation (Kessler et al., 1999; Nock et al., 2008); however, mechanisms driving this transition are unclear, as many identified risk factors are stronger predictors of suicidal ideation compared to suicide attempts (Klonsky & May, 2014; May & Klonsky, 2016). This highlights the need for novel and innovative strategies that more accurately identify at‐risk individuals.

Despite poor predictive utility for future suicidal behaviors, traditional suicide risk assessments, such as the Columbia Suicide Severity Rating Scale (C‐SSRS; Posner et al., 2011), rely on the assessment of the presence and severity (often operationalized as the frequency or intensity of suicidal thoughts and/or the presence of suicidal intent/plans) of suicidal ideation (National Suicide Prevention Lifeline, 2007), sometimes to the extent that patients are deemed not at risk for suicide if they deny suicidal ideation (Silverman & Berman, 2014). However, there are several notable limitations of this approach. Prevalence rates for suicidal ideation are much higher than those for suicidal behaviors (Borges et al., 2012; Nock et al., 2008), suggesting that most individuals who think about suicide do not go on to make a suicide attempt. Indeed, as noted above, the presence and severity of self‐injurious thoughts and behaviors is not a strong predictor of future suicidal thoughts and behaviors (Ribeiro et al., 2016). Moreover, suicidal ideation is transient and fluctuates substantially over time (Kleiman et al., 2017); in many cases, suicidal ideation may not be present when assessed or may be misremembered due to retrospective recall biases (Gratch et al., 2020). Nonetheless, given the centrality of assessing suicidal ideation in determining overall suicide risk, it is unlikely that this approach will, or necessarily should, be abandoned.

Rather than relying solely on the presence and severity of suicidal ideation to identify risk levels, it may be worth examining other characteristics of suicidal ideation (e.g., frequency, duration, controllability) to identify specific features of suicidal ideation that are most predictive of future suicidal behavior. Recent work suggests that there may be different phenotypes of suicidal thinking (Bernanke et al., 2017; Kleiman et al., 2018), characterized by the frequency, intensity, and variability (i.e., fluctuating versus persistent) of suicidal ideation. One study found that individuals with more intense and persistent suicidal thoughts were more likely to have made a recent suicide attempt (Kleiman et al., 2018). Likewise, in samples of adolescents, those who presented to an emergency department following a suicide attempt had longer duration of ideation than those who presented with suicidal ideation only (Negron et al., 1997), and those who reported having a serious wish to die and having planned their most recent suicide attempt for more than an hour were over five times as likely to make another suicide attempt within the 4‐to‐6 year follow‐up period (Miranda, De Jaegere, et al., 2014). Moreover, in an initial prospective examination of characteristics of suicidal ideation among adolescents with lifetime/past‐three‐month suicidal ideation, the frequency of suicidal ideation was uniquely associated with the likelihood of making a suicide attempt at 4‐to‐6‐year follow‐up, above and beyond the recency, duration, and seriousness of ideation and other sociodemographic and clinical characteristics (Miranda, Ortin, et al., 2014). In contrast, studies in adult community and active‐duty military populations have found that low controllability of thoughts across several thought domains, including worry (Gorday et al., 2018), rumination (Rogers et al., 2021), and suicidal ideation (Nock et al., 2018), is a correlate and risk factor for suicidal thoughts and suicide attempts.

Nonetheless, there are several gaps in the literature worth addressing. First, it is unclear if and how different features of suicidal ideation, as assessed by a baseline measure, predict patterns and persistence of suicidal ideation in real time, as assessed by real‐time monitoring methodologies (e.g., ecological momentary assessment [EMA], Shiffman et al., 2008; Stone & Shiffman, 1994). Understanding how baseline measures of suicidal ideation characteristics map onto real‐world trajectories of suicidal ideation is of clinical relevance to mental health practitioners, who may typically only see patients on a weekly basis. Second, characteristics of suicidal ideation measured at baseline and their predictive utility in relation to future suicide attempts have not been examined among recently discharged psychiatric inpatients, who are at substantially elevated risk for suicide attempts (Chung et al., 2017). Replication and extension in this high‐risk population may elucidate when and how certain features of suicidal ideation are worth assessing and directly intervening on. Thus, the present study aimed to examine how baseline characteristics of suicidal ideation (past‐month worst‐point frequency, duration, perceived controllability, and intensity) were associated with (1) the severity and variability of suicidal ideation assessed through EMA; and (2) suicide attempts in the short term (3‐week follow‐up) and longer term (6‐month follow‐up) in a sample of adults who were recently discharged from psychiatric inpatient hospitalization. Although we generally hypothesized that low perceived controllability of suicidal thoughts would be linked to poorer suicide‐related outcomes (Gorday et al., 2018; Nock et al., 2018; Rogers et al., 2021), we made no a priori hypotheses regarding the comparative utility of frequency, duration, or intensity of ideation.

## METHODS

### Participants

Data were collected as part of a larger study examining suicide risk processes in recently psychiatrically hospitalized patients (Armey et al., 2020). Participants were 439 adult psychiatric inpatients recruited from a New England psychiatric hospital. Inclusion criteria were psychiatric hospitalization for suicidal thoughts or behavior (or non‐suicide‐related psychiatric reasons for the psychiatric control condition described below), aged 18–70, English fluency, and comfort with smartphones.

Only participants who completed any EMA during the first 21 days following discharge (*n* = 249) were included in our analyses. Among those included in analyses, 184 (74.2%) were hospitalized for suicidal ideation, 36 (14.5%) for suicide attempts, and 28 (11.3%) for non‐suicidal psychiatric reasons—with the latter also having no lifetime attempts or past‐month ideation. Participants ranged in age from 18 to 69 years (*M* = 40.43; *SD* = 13.30) and were predominantly female (*n* = 136; 55.1%), White (*n* = 222; 91.4%), non‐Hispanic (*n* = 227; 92.3%), and single/never married (*n* = 112, 45.3%). A majority of participants (*n* = 171; 69.0%), 132 of whom were admitted for suicidal ideation and 36 of whom were admitted for suicide attempts, reported a history of at least one lifetime suicide attempt. Detailed sociodemographic characteristics are presented in Table 1.

**TABLE 1:** Sample sociodemographic and clinical characteristics.

Characteristic	*N* (%)
Sex	
Male	111 (44.9)
Female	136 (55.1)
Age [Mean (SD)]	40.43 (13.30)
Race	
White	222 (91.4)
Black/African American	14 (5.8)
Native Hawaiian	0 (0.0)
Asian	4 (1.6)
Native American	3 (1.2)
Ethnicity	
Hispanic/Latino(a)	19 (7.7)
Not Hispanic/Latino(a)	227 (92.3)
Marital status	
Single/Never married	112 (45.3)
Married	54 (21.9)
Separated	7 (2.8)
Divorced	58 (23.5)
Widowed	5 (2.0)
Living together	11 (4.5)
Total household income	
$0–$9999	65 (26.7)
$10,000–$19,999	47 (19.3)
$20,000–$29,999	32 (13.2)
$30,000–$39,999	21 (8.6)
$40,000–$49,999	15 (6.2)
$50,000–$59,999	11 (4.5)
$60,000–$69,999	11 (4.5)
$70,000–$99,999	21 (8.6)
$100,000+	20 (8.2)
Religion	
Roman Catholic	79 (32.1)
Protestant	48 (19.5)
Jewish	2 (0.8)
Other	38 (15.4)
Not affiliated	79 (32.1)
Suicide attempt history	
Lifetime SA	171 (69.0)
Past 3‐month SA	85 (34.3)

Abbreviations: SA, Suicide Attempt; SD, Standard Deviation.

Participants who completed vs. did not complete EMA did not differ with regards to age, race, ethnicity, or reason for hospitalization. However, participants who completed any EMA were significantly more likely to be female (χ^2^[1] = 5.05, *p* = 0.029, *ϕ* = 0.12) and reported a greater number of lifetime suicide attempts (*t*[453] = −2.28, *p* = 0.023, *d* = 0.05; *M*_included_ = 4.48 attempts, *SD* = 9.10 vs. *M*_excluded_ = 2.93, *SD* = 4.52).

A total of 226 participants (90.8%) completed the 3‐week follow‐up assessment, and 204 (81.9%) completed the 6‐month follow‐up. At those time‐points, 34 (15.0%) and 54 (26.5%) participants, respectively, reported the occurrence of a suicide attempt since the previous assessment.

### Procedures

Following psychiatric hospital admission, research staff screened patient charts for inclusion/exclusion criteria. Research staff obtained permission from the inpatient physician to approach patients for recruitment within 72 h of admission. Participants provided informed consent and completed additional eligibility screening, and eligible participants were then recruited into the study. Participants recruited into the study completed a baseline assessment while hospitalized. Baseline entailed an extensive battery of diagnostic interviews, self‐report questionnaires, and lab‐based behavioral tasks; however, the analyses conducted in this paper only focus on baseline and follow‐up assessments of suicidal thoughts and behaviors, as well as EMA data (see Armey et al., 2020, for broader study details). Assessments were administered by research assistants supervised by a licensed clinical psychologist. Beginning on the first day following hospital discharge, participants completed five daily EMA for 21 days. EMA was delivered using the myExperience (Froehlich, 2009), a WinCE application that was later replaced with mEMA (Ilumivu, 2018) due to the discontinuation of WinCE hardware. mEMA is a cross‐platform (iOS and Android) smartphone EMA application. Participants received five random prompts during their typical waking hours, spaced at least one hour apart. Participants had up to 15 min to complete each prompt, receiving two reminders at the 5‐ and 10‐min marks. They were also trained to self‐initiate EMA when they experienced strong suicidal ideation or engaged in suicidal behaviors (e.g., stockpiling pills in preparation for an attempt). When participants endorsed a strong desire to engage in self‐harm or suicidal behavior during EMA, they were automatically prompted by the EMA platform to contact their primary therapist or emergency services or go to their nearest emergency room. Participants returned to the lab for 3‐week and 6‐month follow‐up appointments with assessments comparable to that of baseline. Participants were compensated up to $265 for participation, earning $40 for baseline assessments, $50 for the 3‐week follow‐up assessment, $70 for the 6‐month follow‐up assessment, and $1 for each completed EMA prompt (up to $105).

A total of 235 participants initiated the EMA protocol and were, therefore, included in analyses. Participants completed an average of 26.89 prompts (*SD* = 24.16; range = 1–105), including an average of 5.85 user‐initiated EMAs (*SD* = 9.85; range = 0–91). Regarding signal‐contingent prompts, participants completed, on average, 9.24 days of EMA (*SD* = 6.75) and an average of 2.14 prompts per day (*SD* = 0.80) for a total of 5407 completed assessments, reflecting a signal‐contingent EMA adherence rate of 44.00% per day completed and 42.80% of assessments completed each day. Response rates were not associated with any demographic or clinical characteristics assessed at baseline.

### Measures

#### Baseline and follow‐up suicidal ideation and attempts

Study staff administered items from the Columbia Suicide Severity Rating Scale (C‐SSRS; Posner et al., 2011) interview and Modified Scale for Suicidal Ideation (MSSI; Miller et al., 1986) at baseline and during each follow‐up assessment to assess participants' suicidal ideation and behaviors (lifetime and past‐month at baseline; since last assessment and past‐month at each follow‐up). Present analyses focused on characteristics of suicidal ideation at baseline (specifically, past‐month frequency, duration, intensity, and controllability of thoughts) and their suicide attempts reported at follow‐up. Specifically, drawn from the C‐SSRS, frequency of suicidal ideation was assessed with the question, “*How many times have you had these thoughts?*” with response options ranging from 1 (“*Less than once a week*”) to 5 (“*Many times each day*”). Duration was assessed with, “*When you have the thoughts how long do they last?*” with response options ranging from 1 (“*Fleeting—few seconds or minutes*”) to 5 (“*More than 8 hours/persistent or continuous*”). Perceived controllability was assessed with “*Can you stop thinking about killing yourself or wanting to die if you want to?*” with response options ranging from 1 (“*Easily able to control thoughts”*) to 5 (“*Unable to control thoughts*”). Finally, drawn from the MSSI, intensity of suicidal ideation was assessed with “*When you have thought about suicide, have the thoughts been intense (powerful)? How intense have they been?*” with response options ranging from 0 (“*Very weak”*) to 3 (“*Strong*”). Each suicidal ideation characteristic was assessed regarding the worst point in the past month. Participants also reported their number of suicide attempts in their lifetime at baseline, and since the last assessment at the follow‐ups.

#### EMA‐assessed self‐injurious ideation

Participants were asked “*Since you last completed a questionnaire, have you thought about hurting or killing yourself?*” Those who responded affirmatively were asked about the intensity of their suicidal thoughts (“*Since you last completed a questionnaire, when you have thought about suicide, how intense (powerful) have these thoughts been?*”, rated from 0 [“*None*”] to 4 [“*Strong*”]).

Several indicators were computed to estimate patterns of suicidal and self‐injurious thoughts experienced by each participant: (1) mean scores of suicidal ideation intensity across the 3‐week assessment period; (2) within‐person standard deviations of each participant's suicidal ideation intensity (i.e., average within‐person dispersion) across the assessment period; and (3) the proportion of prompts for which self‐injurious ideation was reported (i.e., relative frequency of self‐injurious ideation). Of note, growth curve models did not identify any significant trajectories of change during the EMA period, supporting the use of these aggregated indices.

### Data analytic strategy

Descriptive statistics and bivariate correlations were first computed to examine the normality and interrelatedness of all variables. Correlations between baseline, 3‐week follow‐up, and 6‐month follow‐up suicidal ideation characteristics (frequency, duration, intensity, controllability) were used to assess the stability/test–retest reliability of these characteristics. Next, a series of linear regression analyses were conducted to examine the unique relations between each past‐month suicidal ideation characteristic at baseline and EMA‐assessed suicidal ideation average intensity, variability, and frequency. Finally, logistic regressions were conducted to examine whether each past‐month suicidal ideation characteristic at baseline uniquely predicted suicide attempts at 3‐week and 6‐month follow‐up. In analyses examining future suicide attempts, we included lifetime suicide attempts in a second model step as a covariate to ensure that suicidal ideation characteristics were relevant above and beyond a suicide attempt history. Missing data from non‐completed prompts were handled via listwise deletion. All data management and analyses were conducted in R.

## RESULTS

### Stability and test–retest reliability of suicidal ideation characteristics

Correlations between suicidal ideation frequency, duration, intensity, and controllability—assessed at baseline, 3‐week follow‐up, and 6‐month follow‐up—are reported in Table 2. Frequency (*r*s = 0.23–0.38), duration (*r*s = 0.24–0.28), and intensity (*r*s = 0.20–0.36) of suicidal ideation were each correlated across time‐points with small to small‐to‐moderate effect sizes, indicating that these characteristics were not stable over time. Perceived low controllability of suicidal ideation, on the other hand, exhibited a moderate correlation from baseline to 3‐week follow‐up (*r* = 0.50), suggesting moderate stability in the short term; however, correlations with the 6‐month follow‐up were small to small‐to‐moderate (*r*s = 0.22 and 0.33) in nature. Low controllability of suicidal ideation was the only baseline characteristic that was associated with all other characteristics of suicidal ideation at baseline and at follow‐up assessments (*r*s = 0.21–0.50), indicating that more uncontrollable suicidal ideation was associated with more frequent, long‐lasting, and intense suicidal ideation.

**TABLE 2:** Descriptive statistics and correlations between suicidal ideation characteristics and suicide attempts across time‐points.

Variable	1	2	3	4	5	6	7	8	9	10	11	12	13	14	15	16	17
1. T1 Frequency	1	0.38***	0.30***	0.42***	0.33***	0.20*	0.11	0.23**	0.10	0.25*	0.08	0.03	0.08	−0.01	0.15*	0.16*	0.16*
2. T1 Duration		1	0.37***	0.45***	0.10	0.24**	0.07	0.22**	0.02	0.19*	0.28**	0.07	0.27*	−0.06	0.00	0.00	0.01
3. T1 Intensity			1	0.49***	0.39***	0.10	0.20*	0.17	0.14	0.20	0.10	0.26*	0.18	−0.02	0.13	0.23**	0.12
4. T1 Control				1	0.28**	0.28**	0.23*	0.50***	0.23**	0.21*	0.24*	0.26*	0.33**	−0.13	0.13	0.18*	0.14
5. T2 Frequency					1	0.29***	0.36***	0.33***	0.35***	0.23*	−0.11	0.03	0.05	0.24**	0.36***	0.38***	0.37***
6. T2 Duration						1	0.32***	0.50***	0.14	−0.01	0.28*	−0.04	0.22	0.14	0.21**	0.16*	0.20*
7. T2 Intensity							1	0.43***	0.25**	−0.06	0.05	0.09	−0.01	0.18	0.23*	0.27**	0.22*
8. T2 Control								1	0.23**	0.15	0.19	0.06	0.22	0.05	0.29***	0.32***	0.29***
9. T2 Attempt									1	−0.08	0.02	0.04	0.01	0.36***	0.36***	0.34***	0.36***
10. T3 Frequency										1	0.17	0.39***	0.41***	0.04	0.06	0.06	0.07
11. T3 Duration											1	0.36***	0.38***	0.12	−0.04	−0.05	−0.06
12. T3 Intensity												1	0.44***	0.15	0.01	0.06	0.01
13. T3 Control													1	0.07	0.07	−0.01	0.06
14. T3 Attempt														1	0.18*	0.15*	0.16*
15. EMA Intensity															1	0.68***	0.98***
16. EMA Variability																1	0.69***
17. EMA Frequency																	1
Mean	3.66	3.08	2.64	3.46	3.29	2.45	2.28	3.15	16%	3.21	2.71	2.31	2.98	27%	0.37	0.45	0.13
SD	1.40	1.46	0.70	1.46	1.51	1.48	0.89	1.56	–	1.52	1.52	0.83	1.59	–	0.72	0.57	0.25
Range	1–5	1–5	0–3	1–5	1–5	1–5	0–3	1–5	0–1	1–5	1–5	0–3	1–5	0–1	0–3.33	0–2.12	0–1
Skewness	−0.54	0.07	−2.16	−0.48	−0.20	0.61	−0.63	−0.16	–	−0.15	0.30	−0.87	0.03	–	2.37	0.91	2.16
Kurtosis	−1.02	−1.35	4.45	−1.11	−1.38	−1.02	0.34	−1.47	–	−1.39	−1.33	−0.32	−1.37	–	4.96	−0.54	3.77

*p < 0.05, **p < 0.01, ***p < 0.001.

### Relations between suicidal ideation characteristics and EMA‐assessed suicidal ideation

Low perceived controllability of suicidal thoughts was uniquely positively associated with greater within‐person variability of suicidal ideation intensity during EMA monitoring (*B* = 0.09, *SE* = 0.04, *p* = 0.049, 95% CI [0.00, 0.17], *f* ^2^ = 0.04). However, contrary to expectations, perceived controllability of suicidal thoughts was unrelated to average levels of suicidal ideation intensity or the frequency of suicidal ideation. Moreover, neither baseline frequency, duration, nor intensity of suicidal ideation was uniquely associated with any EMA‐assessed outcomes. See Table 3 for detailed statistics from all models.

**TABLE 3:** Linear regression analyses examining suicidal ideation characteristics as predictors of EMA‐assessed suicidal ideation characteristics.

Outcome/predictor	B	SE	*p*	95% CI	*f* ^2^
Average intensity					
Frequency	0.03	0.06	0.578	−0.08, 0.15	0.002
Duration	−0.03	0.05	0.495	−0.14, 0.07	0.004
Intensity	0.10	0.11	0.333	−0.11, 0.32	0.001
Controllability	0.04	0.06	0.481	−0.07, 0.15	0.004
Within‐Person variability					
Frequency	0.03	0.04	0.532	−0.06, 0.12	0.003
Duration	−0.06	0.04	0.167	−0.14, 0.02	0.02
Intensity	0.14	0.08	0.084	−0.02, 0.30	0.06
Controllability	0.09	0.04	0.049	0.00, 0.17	0.04
Frequency					
Frequency	0.01	0.02	0.490	−0.03, 0.05	0.004
Duration	−0.01	0.02	0.472	−0.05, 0.02	0.004
Intensity	0.03	0.04	0.456	−0.04, 0.10	0.000
Controllability	0.02	0.02	0.422	−0.02, 0.05	0.003

### Relations between suicidal ideation characteristics and follow‐up suicide attempts

At 3‐week follow‐up, low controllability of suicidal thoughts was uniquely associated with increased likelihood of making a suicide attempt (OR = 1.80, *p* = 0.009, 95% CI [1.19, 2.89]), whereas frequency, duration, and intensity of suicidal thoughts were unrelated to the likelihood of making a suicide attempt. These results were consistent after incorporating lifetime suicide attempts as a covariate. However, at 6‐month follow‐up, no characteristic was uniquely related to likelihood of making a suicide attempt prior to including suicide attempts as a covariate, but low controllability of suicidal thoughts was *negatively* associated with the likelihood of reporting a suicide attempt at 6‐month follow‐up after controlling for lifetime suicide attempts. Detailed statistics of both models are presented in Table 4.^i^

**TABLE 4:** Logistic regression analyses examining suicidal ideation characteristics as predictors of suicide attempts at 3‐week and 6‐month follow‐up.

Outcome/predictor	OR	*p*	95% CI
Three‐week follow‐up			
Frequency	0.93	0.706	0.62, 1.39
Duration	0.73	0.107	0.49, 1.06
Intensity	2.40	0.185	0.77, 10.89
Controllability	1.80	0.009	1.19, 2.89
Three‐week follow‐up			
Frequency	0.93	0.711	0.61, 1.40
Duration	0.73	0.108	0.49, 1.07
Intensity	2.40	0.188	0.75, 10.93
Controllability	1.80	0.012	1.17, 2.94
Lifetime suicide attempt	1.00	0.997	0.30, 3.98
Six‐month follow‐up			
Frequency	1.13	0.498	0.80, 1.61
Duration	0.97	0.878	0.70, 1.35
Intensity	1.42	0.320	0.73, 3.03
Controllability	0.71	0.056	0.50, 1.00
Six‐month follow‐up			
Frequency	1.16	0.402	0.82, 1.67
Duration	0.98	0.925	0.71, 1.37
Intensity	1.26	0.539	0.62, 2.78
Controllability	0.68	0.035	0.46, 0.97
Lifetime suicide attempt	1.82	0.302	0.61, 6.03

## DISCUSSION

This study examined whether different past‐month characteristics of suicidal ideation predicted (1) patterns and persistence of suicidal ideation measured in real time and (2) suicide attempts over subsequent months. We found low to moderate stability of frequency, duration, intensity, and perceived controllability of suicidal ideation across periods of weeks to months. Surprisingly, suicidal ideation characteristics measured at baseline were largely not associated with subsequent patterns of momentary ideation. However, consistent with previous research, low controllability of ideation was associated with both momentary variability in suicidal ideation and increased risk of suicide attempts over the subsequent 3 weeks. These results implicate low perceived controllability of suicidal ideation as a potentially useful marker of short‐term risk for suicidal behavior, with notable implications for suicide risk assessment and monitoring strategies.

Overall, we found that most of the suicidal ideation characteristics we examined (frequency, duration, intensity) were not stable over the subsequent months. This pattern of findings is consistent with theory (Bryan et al., 2020) and research findings (e.g., Gutierrez et al., 2021) underscoring the highly dynamic nature of suicidal ideation. Collectively, evidence suggests that suicide risk assessments conducted at a single point in time are not likely to capture true risk profiles over following months, and thus may have limited prospective clinical utility. There was one notable exception to this pattern of effects, however. Controllability of suicidal ideation showed moderate stability over the 3‐week follow‐up period (*r* = 0.50). Although this effect declined substantively by the 6‐month follow‐up, the perceived uncontrollability of suicidal ideation was moderately associated with greater frequency, intensity, and duration of suicidal ideation cross‐sectionally. Together, these results suggest that low perceived controllability may be a useful indicator of short‐term risk for suicidal ideation, and a marker of the severity of ideation more generally, although additional research is needed to replicate these findings.

An interesting pattern of effects emerged when examining prospective associations between baseline and momentary suicidal ideation characteristics. Overall, characteristics measured at baseline did not generally predict the average intensity or frequency of momentary suicidal ideation intensity over subsequent weeks, despite presumably measuring very similar characteristics of ideation. This finding is particularly surprising given the closeness of the baseline and momentary assessments in time. Methodological factors such as reduced common method variance (Campbell & Fiske, 1959) from using different clinical measures in assessment may have contributed to reduced associations between these measures. These measures may have also captured slightly different aspects of these characteristics of suicidal ideation, as the baseline assessment indexed patient perceptions of recent ideation, whereas momentary assessments indexed metrics of ideation derived from in vivo ratings.

However, it is notable that, among all the characteristics measured, low perceived controllability was the only aspect of suicidal ideation associated with both features of momentary ideation and risk of suicide attempts 3 weeks later. These findings are surprising as the frequency, duration, and intensity of suicidal ideation are often conceptualized in risk assessment as markers of the severity of suicide risk more generally (Posner et al., 2011). That lower suicidal ideation controllability related to greater within‐person variability in ideation, but not other aspects of momentary ideation, is consistent with the idea that individuals with less ability to manage their suicidal thoughts would be more prone to experiencing a wider and less predictable range of ideation severity. Critically, research has found that patients with a higher degree of within‐person variability in suicidal ideation are at elevated risk of suicidal behaviors months later (Bryan et al., 2019). As such, it may be that patients with low controllability have more difficulty coping with suicidal urges, and are thus more prone to engaging in suicidal behaviors. That low perceived controllability was linked with increased risk of suicide attempts in the 3 weeks post‐baseline is consistent with this interpretation, and other research implicating controllability in risk for suicide attempts, albeit retrospectively (Nock et al., 2018; Pearce & Martin, 1993). Importantly, this finding held after controlling for a lifetime history of suicide attempts. This pattern of findings collectively suggests that baseline assessments of low perceived controllability may be a particularly useful marker of short‐term risk for suicidal behavior in particular, and thus may be an important factor for clinicians to assess during patient contacts. However, additional work is needed to replicate and extend these findings to broader samples, particularly since low controllability was negatively linked to suicide attempts 6 months later after controlling for lifetime attempts. This finding may be attributable to statistical artifacts, but it may be reflective of differential relations between perceived cognitive control and suicide risk over time. This, too, deserves attention in future work.

## LIMITATIONS AND FUTURE DIRECTIONS

This study has several limitations worth considering. First, analyses were conducted in a sample of individuals at high risk for suicide, recruited from a psychiatric inpatient sample. Additional research is needed to examine the extent to which our findings generalize to more diverse samples in other clinical and community settings, including individuals who are not engaged in any form of mental health treatment. Second, this study was an observational study, and, therefore, conclusions about the potential causality of effects cannot be drawn. Third, although we examined an array of characteristics of suicidal ideation, other features (e.g., thought content, reactions to suicidal thoughts) were not examined, and may have prospective clinical utility. Likewise, our initial item assessing the presence of ideation in the EMA portion of the study assessed “thoughts of hurting or killing oneself,” precluding a definitive differentiation of non‐suicidal self‐injurious and suicidal self‐injurious thoughts. Although our follow‐up question regarding the intensity of these thoughts specified suicidal thoughts, this may have still impacted our findings. Fourth, our sample was relatively homogeneous in terms of race/ethnicity and was drawn from a limited geographic region. Replication and extension of these findings among individuals from varying locales and minoritized backgrounds is needed for generalizability purposes. Finally, it is worth noting that adherence rates were notably low in the EMA portion of the study, suggesting that these findings may not be representative of true differences in momentary suicidal ideation. This may be attributable to both the acuity of the sample—as high acuity patients may have difficulties completing research protocols, particularly during critical transitions in care (Olfson et al., 2014) and the brief timeframe (i.e., 15 min) in which participants were able to respond to each prompt. Given the importance of understanding suicide risk in individuals following discharge from psychiatric hospitalization, it will be important for future research to identify and implement strategies that reduce barriers to participation in EMA protocols among high‐risk populations.

## CONCLUSIONS

Nevertheless, this study provides an important and novel contribution to the literature about suicide risk assessment. It was the first to examine prospective associations of interview measures of key characteristics of suicidal ideation with subsequent measures of suicide risk, including aspects of momentary suicidal ideation and behavior over shorter (3 weeks) and comparatively longer (6 months), clinically‐relevant (i.e., post‐discharge), time periods. Our results implicate low controllability of suicidal ideation as a potentially useful marker of short‐term risk for suicide attempts. This finding is of high clinical relevance, particularly as perceived controllability is a characteristic of suicidal ideation less frequently assessed in clinical settings than other characteristics of ideation commonly thought to indicate suicidal ideation severity (i.e., frequency, duration, intensity), and as to‐date almost no useful indicators of short‐term suicide attempt risk have been identified that can be assessed via a single item. Although additional research is needed to confirm our findings and examine their applicability to broader clinical samples, our results preliminarily suggest that incorporating a brief assessment of perceived suicidal ideation controllability into clinical contacts may be a low‐burden and readily interpretable means that providers can use to identify patients who may benefit from additional clinical support and/or monitoring to better manage short‐term suicide risk.

## FUNDING INFORMATION

This work was supported by grants from the National Institute of Mental Health (R01MH097741‐03 and R01MH095786‐05), awarded to Dr. Michael Armey. The funder had no further role, and the opinions, interpretations, conclusions, and recommendations expressed do not necessarily represent those of the National Institute of Mental Health.

## CONFLICT OF INTEREST STATEMENT

Dr. Armey is a compensated member of Ilumivu's scientific advisory board and could potentially benefit from the results of this research. No other authors report any conflicts of interest.

## ETHICS STATEMENT

All study procedures were approved by the Butler Hospital Institutional Review Board.

## Data Availability

The data that support the findings of this study are available from the corresponding author upon reasonable request.

## Endnote

^i^
We conducted a sensitivity analysis to examine whether the average intensity and variability of EMA‐assessed suicidal ideation was predictive of suicide attempts at 3‐week and 6‐month follow‐up. Interestingly, EMA‐assessed variability, but not intensity, was associated with increased likelihood of reporting a suicide attempt at 3‐week follow‐up (OR = 3.03, 95% CI [1.33, 7.17]). However, neither EMA‐assessed intensity nor variability predicted the likelihood of reporting a suicide attempt at 6‐month follow‐up (ORs = 1.14 and 1.60, *p*s = 0.140 and 0.739), suggesting that EMA variability may have clinical utility in understanding short‐term, but not longer‐term, suicide risk.

## References

[sltb13103-bib-0001] Armey, M. F. , Brick, L. , Schatten, H. T. , Nugent, N. R. , & Miller, I. W. (2020). Ecologically assessed affect and suicidal ideation following psychiatric inpatient hospitalization. General Hospital Psychiatry, 63, 89–96. 10.1016/j.genhosppsych.2018.09.008 30297091 PMC6581626

[sltb13103-bib-0002] Bernanke, J. A. , Stanley, B. H. , & Oquendo, M. A. (2017). Toward fine‐grained phenotyping of suicidal behavior: The role of suicidal subtypes. Molecular Psychiatry, 22, 1080–1081. 10.1038/mp.2017.123 28607457 PMC5785781

[sltb13103-bib-0003] Borges, G. , Chiu, W. T. , Hwang, I. , Panchal, B. N. , Ono, Y. , Sampson, N. A. , Kessler, R. C. , & Nock, M. K. (2012). Prevalence, onset, and transitions among suicidal behaviors. In Suicide: Global perspectives from the WHO world mental health surveys (pp. 65–74). Cambridge University Press.

[sltb13103-bib-0004] Bryan, C. J. , Butner, J. E. , May, A. M. , Rugo, K. F. , Harris, J. A. , Oakey, D. N. , Rozek, D. C. , & Bryan, A. O. (2020). Nonlinear change processes and the emergence of suicidal behavior: A conceptual model based on the fluid vulnerability theory of suicide. New Ideas in Psychology, 57, 100758. 10.1016/j.newideapsych.2019.100758 PMC7050543 32123464

[sltb13103-bib-0005] Bryan, C. J. , Rozek, D. C. , Butner, J. , & Rudd, M. D. (2019). Patterns of change in suicide ideation signal the recurrence of suicide attempts among high‐risk psychiatric outpatients. Behaviour Research and Therapy, 120, 103392. 10.1016/j.brat.2019.04.001 31104763 PMC7155814

[sltb13103-bib-0006] Campbell, D. T. , & Fiske, D. W. (1959). Convergent and discriminant validation by the multitrait‐multimethod matrix. Psychological Bulletin, 56, 81–105. 10.1037/h0046016 13634291

[sltb13103-bib-0007] Centers for Disease Control and Prevention [CDC] . (2022). WISQARS: Web‐based inquiry statistics query and reporting system . http://www.cdc.gov/ncipc/wisqars/default.htm

[sltb13103-bib-0008] Chung, D. T. , Ryan, C. J. , Hadzi‐Pavlovic, D. , Singh, S. P. , Stanton, C. , & Large, M. M. (2017). Suicide rates after discharge from psychiatric facilities: A systematic review and meta‐analysis. JAMA Psychiatry, 74, 694–702. 10.1001/jamapsychiatry.2017.1044 28564699 PMC5710249

[sltb13103-bib-0009] Franklin, J. C. , Ribeiro, J. D. , Fox, K. R. , Bentley, K. H. , Kleiman, E. M. , Jaroszewski, A. C. , Chang, B. P. , & Nock, M. K. (2017). Risk factors for suicidal thoughts and behaviors: A meta‐analysis of 50 years of research. Psychological Bulletin, 143, 187–232. 27841450 10.1037/bul0000084

[sltb13103-bib-0010] Froehlich, J. (2009). *The MyExperience tool* [Computer software]. http://myexperience.sourceforge.net

[sltb13103-bib-0011] Gorday, J. Y. , Rogers, M. L. , & Joiner, T. E. (2018). Examining characteristics of worry in relation to depression, anxiety, and suicidal ideation and attempts. Journal of Psychiatric Research, 107, 97–103. 30384092 10.1016/j.jpsychires.2018.10.004

[sltb13103-bib-0012] Gratch, I. , Choo, T. , Galfalvy, H. , Keilp, J. G. , Itzhaky, L. , Mann, J. J. , Oquendo, M. A. , & Stanley, B. (2020). Detecting suicidal thoughts: The power of ecological momentary assessment. Depression and Anxiety, 38, 8–16. 10.1002/da.23043 32442349

[sltb13103-bib-0013] Gutierrez, P. M. , Joiner, T. , Hanson, J. , Avery, K. , Fender, A. , Harrison, T. , Kerns, K. , McGowan, P. , Stanley, I. H. , Silva, C. , & Rogers, M. L. (2021). Clinical utility of suicide behavior and ideation measures: Implications for military suicide risk assessment. Psychological Assessment, 33, 1–13. 10.1037/pas0000876 33180522

[sltb13103-bib-0014] Kessler, R. C. , Borges, G. , & Walters, E. E. (1999). Prevalence of and risk factors for lifetime suicide attempts in the National Comorbidity Survey. Archives of General Psychiatry, 56, 617–626. 10.1001/archpsyc.56.7.617 10401507

[sltb13103-bib-0015] Kleiman, E. M. , Turner, B. J. , Fedor, S. , Beale, E. E. , Huffman, J. C. , & Nock, M. K. (2017). Examination of real‐time fluctuations in suicidal ideation and its risk factors: Results from two ecological momentary assessment studies. Journal of Abnormal Psychology, 126, 726–738. 10.1037/abn0000273 28481571

[sltb13103-bib-0016] Kleiman, E. M. , Turner, B. J. , Fedor, S. , Beale, E. E. , Picard, R. W. , Huffman, J. C. , & Nock, M. K. (2018). Digital phenotyping of suicidal thoughts. Depression and Anxiety, 35, 601–608. 10.1002/da.22730 29637663

[sltb13103-bib-0017] Klonsky, E. D. , & May, A. M. (2014). Differentiating suicide attempters from suicide ideators: A critical frontier for suicidology research. Suicide and Life‐threatening Behavior, 44, 1–5. 10.1111/sltb.12068 24313594

[sltb13103-bib-0018] May, A. M. , & Klonsky, E. D. (2016). What distinguishes suicide attempters from suicide ideators? A meta‐analysis of potential factors. Clinical Psychology: Science and Practice, 23, 5–20. 10.1111/cpsp.12136

[sltb13103-bib-0019] Miller, I. W. , Norman, W. H. , Bishop, S. B. , & Dow, M. G. (1986). The modified scale for suicidal ideation: Reliability and validity. Journal of Consulting and Clinical Psychology, 54, 724–725. 10.1037//0022-006x.54.5.724 3771893

[sltb13103-bib-0020] Miranda, R. , De Jaegere, E. , Restifo, K. , & Shaffer, D. (2014). Longitudinal follow‐up study of adolescents who report a suicide attempt: Aspects of suicidal behavior that increase risk of a future attempt. Depression and Anxiety, 31, 19–26. 10.1002/da.22194 24105789

[sltb13103-bib-0021] Miranda, R. , Ortin, A. , Scott, M. , & Shaffer, D. (2014). Characteristics of suicidal ideation that predict the transition to future suicide attempts in adolescents. Journal of Child Psychology and Psychiatry, and Allied Disciplines, 55, 1288–1296. 10.1111/jcpp.12245 24827817 PMC4821401

[sltb13103-bib-0022] National Suicide Prevention Lifeline . (2007). Suicide risk assessment standards . https://suicidepreventionlifeline.org/wp‐content/uploads/2016/08/Suicide‐Risk‐Assessment‐Standards‐1.pdf

[sltb13103-bib-0023] Negron, R. , Piacentini, J. , Graae, F. , Davies, M. , & Shaffer, D. (1997). Microanalysis of adolescent suicide attempters and ideators during the acute suicidal episode. Journal of the American Academy of Child and Adolescent Psychiatry, 36, 1512–1519. 10.1016/S0890-8567(09)66559-X 9394935

[sltb13103-bib-0024] Nock, M. K. , Borges, G. , Bromet, E. J. , Alonso, J. , Angermeyer, M. , Beautrais, A. , Bruffaerts, R. , Chiu, W. T. , de Girolamo, G. , Gluzman, S. , de Graaf, R. , Gureje, O. , Haro, J. M. , Huang, Y. , Karam, E. , Kessler, R. C. , Lepine, J. P. , Levinson, D. , Medina‐Mora, M. E. , … Williams, D. (2008). Cross‐national prevalence and risk factors for suicidal ideation, plans and attempts. The British Journal of Psychiatry, 192, 98–105. 10.1192/bjp.bp.107.040113 18245022 PMC2259024

[sltb13103-bib-0025] Nock, M. K. , Millner, A. J. , Joiner, T. E. , Gutierrez, P. M. , Hwang, I. , King, A. , Naifeh, J. A. , Sampson, N. A. , Zaslavsky, A. M. , Stein, M. B. , Ursano, R. J. , & Kessler, R. C. (2018). Risk factors for the transition from suicide ideation to suicide attempt: Results from the Army study to assess risk and resilience in Servicemembers (Army STARRS). Journal of Abnormal Psychology, 127, 139–149. 10.1037/abn0000317 29528668 PMC5851467

[sltb13103-bib-0026] Olfson, M. , Marcus, S. C. , & Bridge, J. A. (2014). Focusing suicide prevention on periods of high risk. JAMA, 311, 1107–1108. 10.1001/jama.2014.501 24515285

[sltb13103-bib-0027] Pearce, C. M. , & Martin, G. (1993). Locus of control as an indicator of risk for suicidal behaviour among adolescents. Acta Psychiatrica Scandinavica, 88, 409–414. 10.1111/j.1600-0447.1993.tb03482.x 8310847

[sltb13103-bib-0028] Posner, K. , Brown, G. K. , Stanley, B. , Brent, D. A. , Yershova, K. V. , Oquendo, M. A. , Currier, G. W. , Melvin, G. A. , Greenhill, L. , Shen, S. , & Mann, J. J. (2011). The Columbia–suicide severity rating scale: Initial validity and internal consistency findings from three multisite studies with adolescents and adults. The American Journal of Psychiatry, 168, 1266–1277. 10.1176/appi.ajp.2011.10111704 22193671 PMC3893686

[sltb13103-bib-0029] Ribeiro, J. D. , Franklin, J. C. , Fox, K. R. , Bentley, K. H. , Kleiman, E. M. , Chang, B. P. , & Nock, M. K. (2016). Self‐injurious thoughts and behaviors as risk factors for future suicide ideation, attempts, and death: A meta‐analysis of longitudinal studies. Psychological Medicine, 46, 225–236. 10.1017/S0033291715001804 26370729 PMC4774896

[sltb13103-bib-0030] Rogers, M. L. , Gorday, J. Y. , & Joiner, T. E. (2021). Examination of characteristics of ruminative thinking as unique predictors of suicide‐related outcomes. Journal of Psychiatric Research, 139, 1–7. 10.1016/j.jpsychires.2021.05.001 33992843

[sltb13103-bib-0031] Shiffman, S. , Stone, A. A. , & Hufford, M. R. (2008). Ecological momentary assessment. Annual Review of Clinical Psychology, 4, 1–32. 10.1146/annurev.clinpsy.3.022806.091415 18509902

[sltb13103-bib-0032] Silverman, M. M. , & Berman, A. L. (2014). Suicide risk assessment and risk formulation part I: A focus on suicide ideation in assessing suicide risk. Suicide & Life‐Threatening Behavior, 44, 420–431. 10.1111/sltb.12065 25250407

[sltb13103-bib-0033] Stone, A. A. , & Shiffman, S. (1994). Ecological momentary assessment in behavioral medicine. Annals of Behavioral Medicine, 16, 199–202.

[sltb13103-bib-0035] World Health Organization [WHO] . (2021). Suicide mortality rate (per 100,000 population) . https://www.who.int/data/gho/data/indicators/indicator‐details/GHO/suicide‐mortality‐rate‐(per‐100‐000‐population)

